# Antiviral activity of chrysin and naringenin against porcine epidemic diarrhea virus infection

**DOI:** 10.3389/fvets.2023.1278997

**Published:** 2023-12-07

**Authors:** Mengfei Gong, Xuemei Xia, Dishi Chen, Yupeng Ren, Yutong Liu, Hua Xiang, Xiaohuan Li, Yupeng Zhi, Yu Mo

**Affiliations:** ^1^College of Animal Science and Veterinary Medicine, Southwest Minzu University, Chengdu, China,; ^2^Center for Animal Disease Prevention and Control, Chengdu, China; ^3^Agricultural and Rural Bureau of Shizhong District, Leshan, China

**Keywords:** porcine epidemic diarrhea virus, natural compounds, chrysin, naringenin, antiviral activity

## Abstract

Porcine epidemic diarrhea virus (PEDV) is one of the critical pathogens causing diarrhea in piglets and has caused huge economic losses to the swine industry in worldwide. However, there is currently no effective therapeutic medication available for the treatment of PEDV. Natural compounds are a hot topic for researching and screening antiviral lead compounds due to their abundant sources, varied activities, and low toxicity. In this study, a total of 6 compounds from different plant sources were selected for *in vitro* anti-PEDV screening, including chrysin, naringenin, soy isoflavone, glycyrrhetinic acid, oleanolic acid, and geniposide. Then two active compounds, chrysin and naringenin, were further evaluated on PEDV infected cells at different stage. And the anti-PEDV mechanism was analyzed by molecule docking and molecular dynamics. The results showed that both chrysin and naringenin showed the most significant anti-PEDV activity by increasing the cell viability and decreasing the virus copy number. Both natural compounds could inhibit viral titer, mRNA and protein levels in the prophylactic and post-viral entry stages of PEDV infection. Furthermore, chrysin and naringenin mainly interacted with viral replicase proteins such as 3CLpro and PLP-2 through hydrogen bonds and hydrophobic forces. The complexes formed by chrysin and naringenin with the two PEDV replication proteases had high stability. These results suggested that chrysin and naringenin may exert antiviral effects by interacting with the virus 3CLpro protein or PLP2 protein, thereby affecting their role in the formation of PEDV non-structural proteins or interfering with virus replication. This study lays the foundation for developing chrysin and naringenin as novel anti-PEDV therapeutic drugs.

## 1 Introduction

Porcine epidemic diarrhea (PED), one of the acute enteritis infections, is characterized by vomiting and watery diarrhea (1) and caused by the porcine epidemic diarrhea virus (PEDV) (genus alpha coronavirus, the order *Nidovirales*) and has always been a limiting factor for the healthy development of the pig breeding industry (2). It usually threatened neonates’ survival rates, leading to the growth retardation of weaned piglets (3), which caused enormous economic losses for the global swine industry and international trade (4). PEDV is very destructive to the intestinal barrier of piglets and has a high mortality rate of 95% for those under 2 weeks of age piglets (5). The obvious atrophy of the intestinal villi and massive enterocyte shedding in the small intestine of the PEDV-infected model was observed (6). For the last decade, PEDV has been circulated in the swine population worldwide (7). The survey implies that PED has been a significant disease in the North American and European swine industry during 2013–2017. And >3,750 premises in 39 states in America were confirmed as positive for the virus (8). In Asia, PEDV has also been a pandemic in China (9), Japan (10), Korea (11), and Vietnam (12). For instance, a cross-sectional survey showed that the percentage of PEDV-positive farms in northern Vietnam was 30.9% (101/327) during 2018–2019. And the highest proportion of PEDV-positive farms was 70% (7/10) among nucleus production type farms (*p* < 0.05) (13). Nguyen reported that there were at least three genotypes (North American (NA), S INDEL, and Asian non-S INDEL) circulating in Japan from 2013 to 2016 (10). In China, PEDV had been circulated in at least 8 provinces during 2018–2021, with an average positive rate of 56.09%. And the proportion reached 56.44% (740/1311) in 2021 (9). It has been a main etiology for gastrointestinal infectious diseases of pigs, causing serious economic losses (14).

Many kinds of commercial live attenuated vaccines of PEDV have been administrated to prevent infection. However, the efficacy of protection against heterologous PEDV field strains was unsatisfactory, attributed to the viral genetic diversity and variability (15). Furthermore, there are no specific medications against PEDV. However, the study of anti-PEDV drugs is still in its initial stage. Therefore, developing and screening high-efficiency, low-toxicity, and low-residue drugs with targeted treatment for PEDV are the actual demand for veterinary usage in clinics and the important direction of future studies (16).

In recent years, the antiviral effects of natural compounds, especially plant extracts and Chinese herbal medicines, had been gradually revealed. Due to its advantages of rich sources, unique chemical structure, and diverse activities, natural compounds have gradually been a hot topic in the development of new drugs (17). Flavonoids are a kind of polyphenols mainly derived from plants. They comprise two benzene rings with phenolic hydroxyl groups connected through the central three-carbon atom (18). Studies have shown that many flavonoids have good antiviral activity. For example, xanthohumol can induce the expression level of HMOX1 to increase dose-dependent, thus significantly inhibiting the proliferation of porcine reproductive and respiratory syndrome virus (PRRSV) in Marc-145 cells and PAM cells. And by activating the Nrf2-HMOX1 pathway, it can regulate the antioxidant effect of Nrf2 and alleviate the oxidative stress damage caused by PRRSV stimulation (19). Dewi et al. (20) found that the flavonoid derivative quercetin had an inhibitory effect on the dengue virus. The quercetin CC50 was 217.113 μg/mL in human Huh 7 it-1 cell line, and the IC50 against DENV-2 was 18.41 μg/mL, with an SI value of 11.8. In addition, quercetin could inhibit the NF-κB production of NLRP3 inflammasomes and IL-1β and inhibit the inflammatory response by interfering with the NF-κB pathway. Thereawatnasirikul et al. (21) found that luteolin and isoginkgetin can significantly inhibit the proliferation of foot-and-mouth disease virus (FMDV) on BHK-21 cells. The SI values of luteolin and isoginkgetin with the range of 10.00–51.81 μM in viral and post-viral entry experiments were greater than 4, which is considered as potent antiviral agents; besides, luteolin can also inhibits Japanese Encephalitis virus and Ebola virus. However, limited research exists regarding the screening and research of natural flavonoid compounds resistant to PEDV. The mechanism of the antiviral activities of flavonoids is still unclear. And further studies in t the relevant fields will provide important assistance in the prevention and treatment of pig viral diarrhea (22).

In this study, the inhibitory effect of chrysin and naringenin on PEDV was confirmed for the first time, and the mechanism of the antiviral effect of chrysin and naringenin was further investigated to determine the main stages of their action. The interaction of the two natural compounds with PEDV replication related proteins was predicted and analyzed, laying the foundation for further developing novel anti-PEDV therapeutic drugs with chrysin and naringenin and providing a theoretical basis.

## 2 Materials and methods

### 2.1 Cell and virus

Vero E6 cells were maintained in American Type Culture Collection, PEDV strain DY (field isolate) and CV777 strain are preserved by the College of Southwest University for Nationalities Laboratory of Animal Medical of China.

### 2.2 Reagents and antibodies

The mouse anti-PEDV-S monoclonal antibody and anti-PEDV-N polyclonal antibody were presented by Professor Zhiwen Xu of Sichuan Agricultural University. The horseradish peroxidase (HRP)-labeled secondary antibodies goat anti-mouse IgG/HRP and goat anti-rabbit IgG/HRP were purchased from Southern Biotech. The GAPDH primary antibody was used as the internal reference. The chemicals used were reagent grade.

### 2.3 Cytotoxicity assay

To determine the toxicity of native compounds on Vero E6 cells, MTT assay was used; 1 × 10^6^ cells were seeded onto 96-well plates and incubated at 37°C in a 5% CO_2_ humidified incubator for 24 h. Then, different concentrations of compounds in DMEM were added to cells and incubated for another 48 h. Set up 5 repeated tests for each concentration. MTT solution (10 μL per well) was added and incubated for another 4 h at 37°C, discarding the native compounds and using dimethyl sulfoxide (DMSO) to dissolve products for 10 min at 37°C. Microplate reader (BioTek, Winooski, VT, United States) was used to measure the 96-well plate at 570 nm, and the cell viability (%) was calculated by OD values (23). Taking the drug concentration as the abscissa and the percentage of cell viability as the ordinate, the dose-dependent curve of cell viability was drawn by Graphpad 5.0 software and regression was performed to calculate the 50% cytotoxity concentration (CC50) (24). The percentage of relative cell viability was calculated according to the formula:


$$\mathrm{Relative}\;\mathrm{cell}\;\mathrm{viability}\left(\%\right)=\frac{\mathrm{Compounds}\;\mathrm{group}\;\mathrm{cell}\;OD\;\mathrm{value}}{\mathrm{Cell}\;\mathrm{control}\;\mathrm{group}\;OD\;\mathrm{value}}$$

### 2.4 Antiviral activity assay

The Vero E6 cells were seeded in 96-well plates and incubated under 5% CO_2_ at 37°C into an 80%–90% confluent monolayer. Add 30 μL of 100 TCID_50_ PEDV into each well, and after incubation for 1 h, add 150 μL of compounds with different concentrations in the nontoxic concentration range, and set up DMEM blank control group and virus control group respectively, and set up 5 repeated tests for each concentration. After 48 h of culture, the OD value was detected by MTT assay. Taking the drug concentration as the abscissa and the virus inhibition rate as the ordinate, the dose-dependent curve of the virus inhibition rate was drawn by Graphpad 5.0 software, and the regression was carried out to calculate the IC50 and therapeutic index (SI) of the virus inhibition rate (%) as previously described (25). Calculate the inhibition rate of virus according to the formula:


$$\mathrm{Virus}\;\mathrm{inhibition}\;\mathrm{rate}\;\left(\%\right)=\frac{\begin{array}{l} \mathrm{Compounds}\;\mathrm{group}\;\mathrm{cell}\;OD\;\mathrm{value}\\ -\mathrm{virus}\;\mathrm{group}\;OD\;\mathrm{value} \end{array}}{\begin{array}{l} \mathrm{Cell}\;\mathrm{control}\;\mathrm{group}\;OD\;\mathrm{value}\\ -\mathrm{virus}\;\mathrm{group}\;OD\;\mathrm{value} \end{array}}\times 100\%$$

### 2.5 Indirect immunofluorescence assay

Cells cultured on 6-well plates were divided into experimental group, blank control group and virus control group. They were washed with precooled PBS for three times, then added with 1 mL of 80% acetone (diluted by PBS), immediately placed at 4°C and kept in the dark for 10 min; after washing with PBS for three times, the cells were blocked in TBST containing 3% bovine serum albumin (BSA) for 30 min at room temperature. Then, the cells were washed for three times, and incubated with anti-PEDV S protein antibody for 45 min at room temperature in the dark. After washing three times with PBS, the cells were incubated with FITC labeled goat anti-mouse secondary antibody at 37°C for 45 min. After five washes, DAPI was added and stained at room temperature for 5 min, and the viral loads were observed by fluorescence microscope as previously described (26).

### 2.6 Virus infectivity titer

PEDV was diluted 10 times with the serum-free DMEM and inoculated on the monolayer Vero cells in 96-well plates at 37°C with 5% CO_2_ for 1 h, the cells were replaced with serum-free DMEM. Plates were incubated at 37°C for 48 hpi to 72 hpi, and the cytopathic effect was observed under an inverted microscope. TCID_50_ was calculated according to Reed and Muench formula as previously described (27).

### 2.7 Quantitative RT-PCR

The RT-PCR reactions were performed using a PrimeScript RT Reagent Kit (TaKaRa, Dalian, China). Then the expression of N protein at the transcription level in chrysin, naringenin treatment group and virus group was detected by absolute quantitative PCR. Synthesis according to references using gene-specific primers and probes, according to that synthesis of reference (28) as follow: (PEDV-N-F: CGCAAAGACTGACACTAA, PEDV-N-R: CGCAAAGACTGACACTAA, probe: TGTTGCCATGCCAGACTCCTCC-TAMAR), linear relationship curve expression between copy number and Ct value: Ct = −3.221 × 1 g CopyNumber +36.702. Substituting the Ct value of the PCR assay into the standard curve equation, the initial copy number of clinical sample DNA could be calculated (29).

### 2.8 Time course inhibition assay

**Figure 1:**
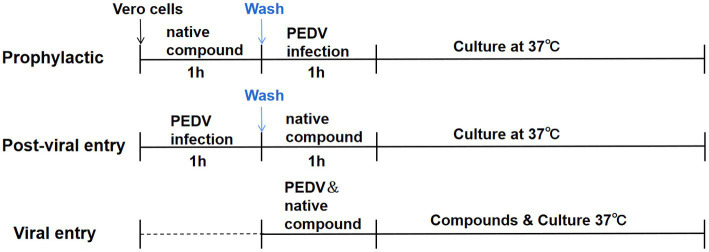
Time-of-addition schematic. Vero E6 cells were infected with PEDV for 1 h (0 to 1 h), and cells were treated with chrysin (50, 25, 12.5 μg/mL) and naringenin (25, 12.5, 6.25 μg/mL) at different times of infection, designated prophylactic, post-viral entry or viral entry.

A time-of-addition experiment was performed, as shown in the timeline schematic, to assess the inhibitory effect of chrysin and naringenin on the PEDV replication cycle (Figure 1). These cells were divided into natural compounds treatment group, virus control group and blank control group. Vero cells were grown in 12-well plates to confluence and then infected with PEDV for 1 h at 37°C (30). Chrysin or naringenin was added before, during, or after PEDV infection.

The prophylactic activity of chrysin and naringenin were evaluated by incubating compounds on the cells for 2 h before viral inoculation, respectively. In this procedure, the intracellular activities of the compounds against PEDV were examined. Thus, cellular uptake of the tested compound was necessary. The cells were incubated with the compounds in different concentration at 37°C for 1 h. Subsequently, the supernatant was suck off, and the cells were washed once with PBS. Then, the virus (100 TCID_50_/well) was incubated at 37°C for 24 h.

To examine whether the compounds can prevent the virus from adhering to or infecting host cells through direct interaction with PEDV, the viral entry experiment was conducted. The PEDV at 100 TCID_50_ was mixed with different concentrations of tested compounds prior to incubation with the cells at 37°C for 1 h. The supernatant was then replaced with fresh DEME and the cells were incubated at 37 for 24 h.

In the post-viral entry experiment, the effects of the compounds on the virus after the entry step were examined. The cells were incubated with PEDV (100 TCID_50_/well) at 37°C for 1 h for viral adsorption. Then, the cells were treated with different concentrations of tested compounds as mentioned above at 37°C for 24 h. In all experiments, viral reduction was analyzed using RT-PCR and western blot, respectively.

### 2.9 Western blot assay

The cell samples to be collected were lysed with lysate and heated at 95°C for 5 min. After SDS-PAGE, the target protein was cut and transferred to nitrocellulose (NC) membrane, adding blocking solution (TBST solution containing 2% BSA), and blocking for 30 min at room temperature. Primary antibodies (rabbit anti-PEDV-N polyclonal antibody, rabbit anti-actin monoclonal antibody) were added and blocked overnight at 4°C. After the end of anti-incubation, the membrane was washed three times with TBST, and the secondary antibody of sheep anti-rabbit labeled with horseradish peroxidase (HRP) was shaken for 1–1.5 h at room temperature on a shaker. The membrane was then washed 3 to 5 times with TBST (31). The target protein bands were observed and detected by ECL enhanced chemiluminescence system. β-actin was used as an internal control.

### 2.10 Molecular docking

#### 2.10.1 Protein preparation

The 3D structure of S protein, papain-like protease2 (PLP-2) and 3C-likeprotease (3CLpro), were obtained by searching the PEDV protein database on the Uniprot website.^1^ The crystal structures of the target proteins were visualized and preprocessed by using PyMOL software for water molecules removal, hydrogenation, and optimization of amino acids (32). The energy of the proteins was optimized by using Autodock Vina and subsequently converted to the pdbqt format.

#### 2.10.2 Ligand preparations

The 3D chemical structures of chrysin in an SDF format (PubChem CID, 5281607) were retrieved from the chemical database PubChem available on the NCBI website^2^ (33). The OpenBabel online software^3^ was used to convert the SDF files of the ligands to PDB format. The ligands were prepared by detecting the torsion root, correcting the torsion angles, assigning charges, optimizing using UFF (Universal force field), and finally converting them to pdbqt format to generate 3D atomic coordinates of the molecules (34).

#### 2.10.3 Receptor grid generation and molecular docking

Molecular docking of selected flavonoids to major proteases and S proteins was performed using Autodock Vina, version 1.2.0. PDB files of target proteins and small molecule ligands were loaded into Autodock Vina and converted to pdbqt. format file, according to the active site of the protein molecule, generates a Grid box and the exhaustiveness was set to 100 (35). After completion of the docking algorithm, the ligand-protein complexes that have the best conformation and lowest binding energy were selected and visualized in Pymol for their conventional and hydrophobic interactions.

### 2.11 Molecular dynamics studies

Molecular dynamics (MD) simulations were used to study the stability of ligand-protein complexes, and CABS-flex 2.0 generated models representing their conformational structures within 10 nm, with fluctuation plots on each chain representing the number of amino acid residues versus the change in RMSF in nanometers. The ligand-protein complex with the best conformation and lowest binding energy was submitted to the CABS-flex 2.0 web server using default parameters (36).

### 2.12 Statistical analyses

All statistical analyses were performed using GraphPad Prism (version 5.0, GraphPad Software, San Diego, CA, United States), and the data were expressed as the mean ± standard deviation. SPSS Statistics v20.0 software (IBM Corp.) was used for the statistical analysis. The significance within groups were determined by one-way or two-way analysis of variance. ^*^p < 0.05, ^**^p < 0.01, and ***p < 0.001 were considered to be statistically significant at different levels.

## 3 Results

### 3.1 Cytotoxicity and antiviral activity of the flavonoids compounds

**Figure 2:**
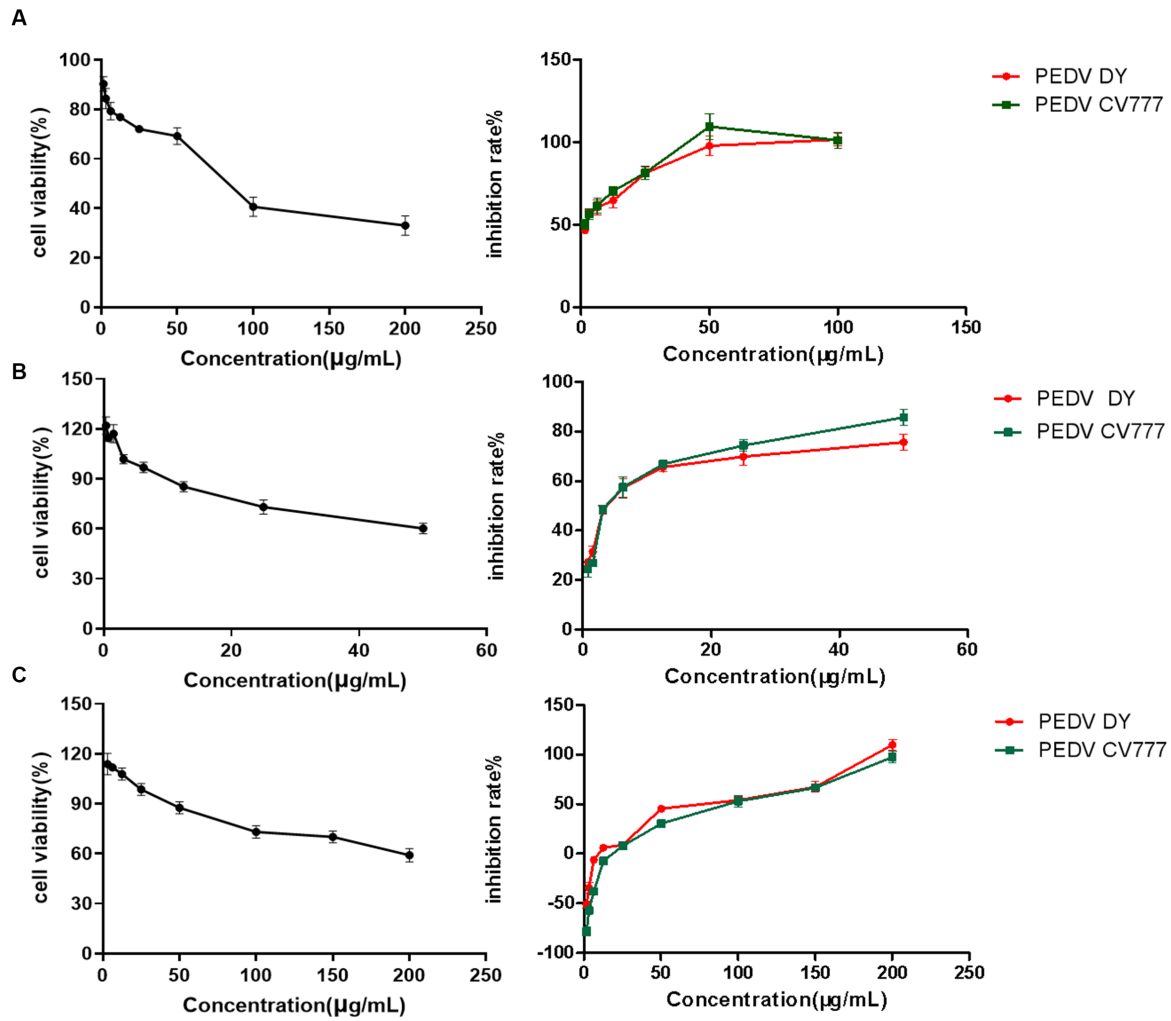
The cytotoxic effect and cytotoxic concentration of natural flavonoid compounds and a dose-response curve showing the inhibition effect of compounds treatment on infection of PEDV live virus in Vero E6 cells. **(A)** Chrysin. **(B)** Naringenin. **(C)** Ribavirin.

The dose-concentration dependence curves of several natural flavonoid compounds were regressed. The half-cytotoxic concentration (CC50) values were calculated according to the inhibitory percentage of the tested compounds at various concentrations on the viability of cells (Figures 2A–C). The results showed that several natural flavonoid compounds had different cytotoxicity to Vero E6 cells, and cell viabilities were decreased in a dose-dependent manner with the increase of the concentration of the compounds. The cytotoxicity of chrysin was the smallest with the CC50 of 83.56 ± 2.12 μg/mL. The oleanolic acid and geniposide exhibited greater cytotoxicity on Vero E6 cells with the CC50 of 4.054 ± 3.91 μg/mL and 10.38 ± 2.03 μg/mL, respectively. Besides, other compounds exhibited less cytotoxicity.

The anti-PEDV activity assessment of the natural compounds showed that chrysin and naringenin had significant inhibition effects on proliferation of two PEDV strains with lower IC50 values (Table 1). The SI values of chrysin and naringenin against two PEDV strains were significantly higher than that of the ribavirin treatment group (*p* < 0.05). The results indicated that both chrysin and naringenin have good inhibition effects against PEDV *in vitro*. Besides, the IC50 values of quercetin, soybean isoflavone, glycyrrhetinic acid, oleanolic acid and geniposide against PEDV were greater than 100 μg/mL, which showed a weak inhibition effect.

**Table 1:** Cytotoxicity to Vero E6 cells, and anti-PEDV activity of phytochemical compounds.

Compounds	CC50 (μg/mL)	PEDV DY		PEDV CV777	
		IC50 (μg/mL)	SI	IC50 (μg/mL)	SI
Chrysin	83.56 ± 2.12	2.484 ± 0.59	33.63	2.192 ± 2.25	38.1
Naringenin	61.86 ± 0.97	4.505 ± 2.25	13.73	4.392 ± 0.96	14.08
Soy isoflavone	53.03 ± 2.30	>100	—	>100	
Glycyrrhetinic acid	17.40 ± 4.61	>100	—	>100	
Oleanolic acid	4.054 ± 3.91	>100	—	>100	
Geniposide	10.38 ± 2.03	>100	—	>100	
Ribavirin	249 ± 4.20	71.39 ± 3.78	3.49	87.99 ± 3.78	2.82

### 3.2 Observation of change in the amount of PEDV after drug administration by indirect immunofluorescence assay

**Figure 3:**
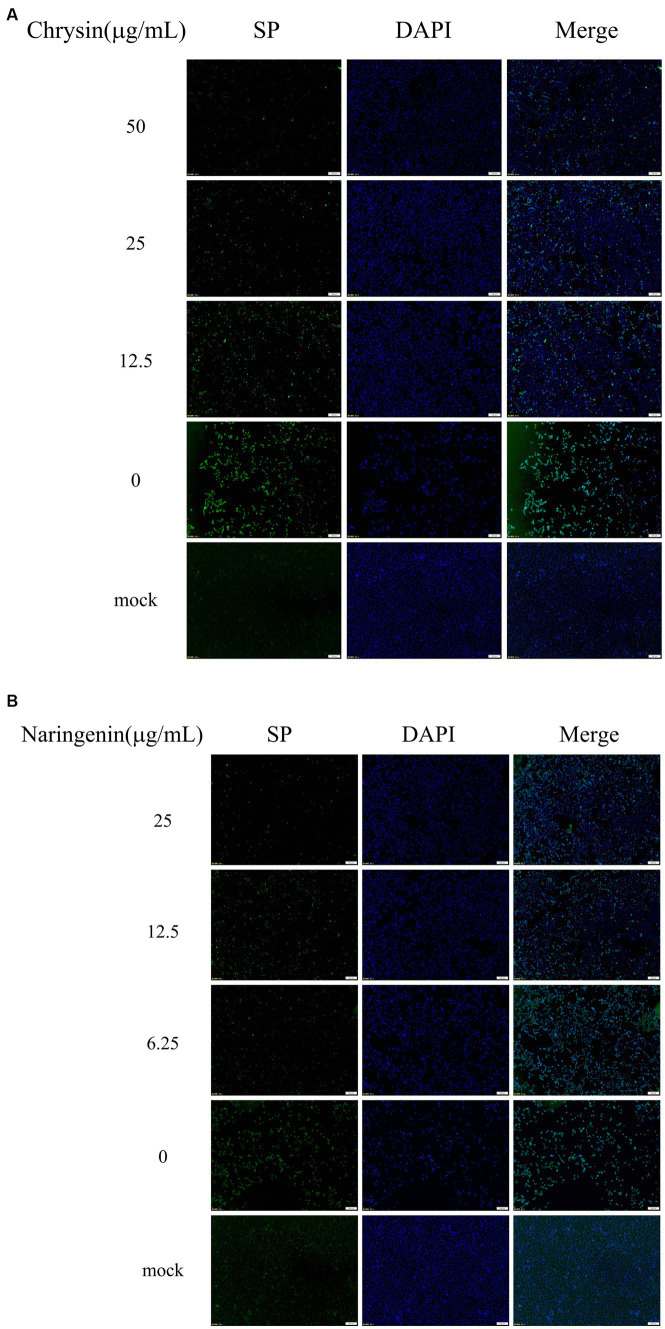
The viral loads were detected by indirect immunofluorescence assay (IFA). Scale bar: 100 μm. **(A)** Chrysin. **(B)** Naringenin.

Vero E6 cells were stained for fluorescence 48 h after infection with PEDV. The results showed that no visible green fluorescence in the negative group under the microscope. The nuclear blue fluorescence of cells in the PEDV control group was significantly diminished, and a substantial number of cells seemed to shed and perish. The green fluorescence of viral proteins was diminished to varying extents in the cells of the chrysin and naringenin-treated groups. The reduction in fluorescence was directly related to the concentration of the compounds, with the strongest impact observed at higher doses (Figures 3A,B). It was indicated that both compounds had a significant inhibitory effect on PEDV.

### 3.3 Viral quantification by qPCR

**Figure 4:**
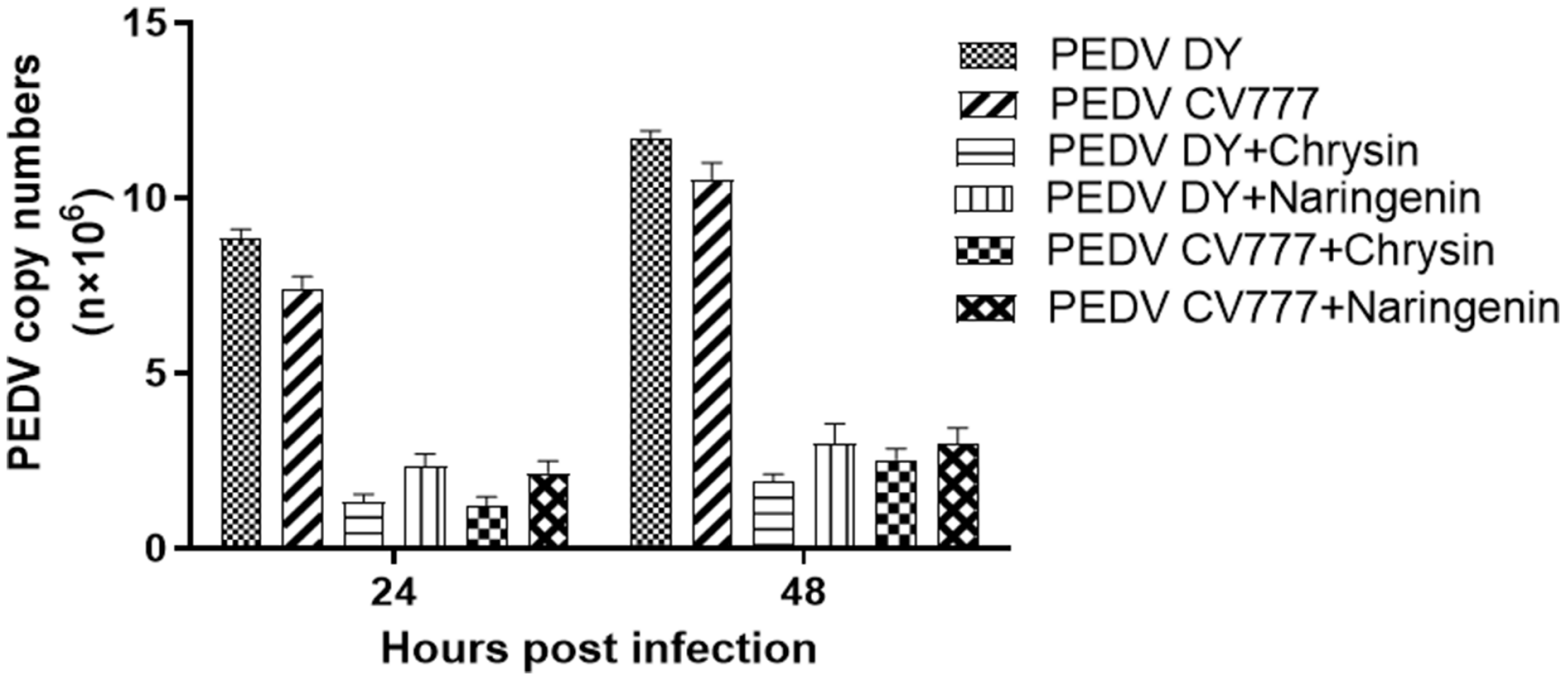
Nucleic acid copy number changes in groups treated with different natural compounds.

To preliminary evaluate the effects of chrysin and naringenin on PEDV replication. The infected cells were treated with two candidate compounds (chrysin 50 μg/mL, naringenin 12.5 μg/mL). Then, the copy number of the virus at different time points was detected by qPCR with Taqman probe. The results showed that the virus copy numbers in the chrysin and naringenin-treated groups were significantly lower than in the control group (*p* < 0.001) at 24 hpi and 48 hpi post-infection. Moreover, the copy number of the chrysin treatment group was lower than that of the naringenin treatment group (*p* < 0.05) (Figure 4). It was indicated that chrysin and naringenin could effectively inhibit the proliferation of PEDV, and chrysin had a greater effect than naringenin.

### 3.4 Virus titration

**Figure 5:**
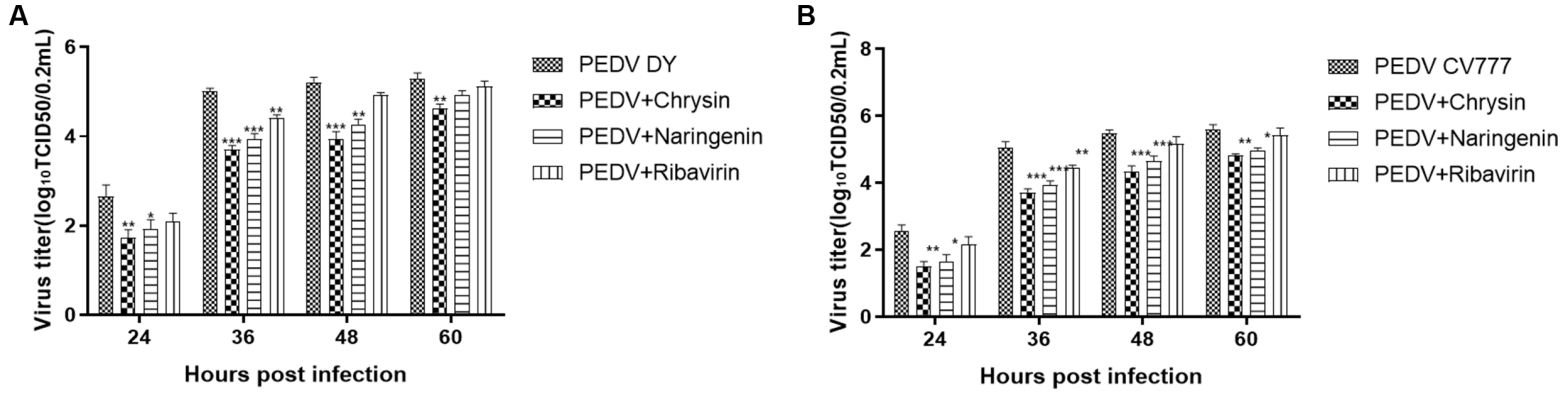
Determination of viral titer after the addition of natural compounds. Ribavirin was used as the treatment control. The results were expressed as TCID_50_/0.2 mL. The titers from the three independent experiments were displayed as mean SEMs. **(A)** PEDV DY. **(B)** PEDV CV777.

The cells treated with natural compounds were observed with an inverted microscope at different times. The virus titers were calculated using the Reed-Muench method and expressed as TCID_50_/0.2 mL. The results showed that chrysin, naringenin, and ribavirin could inhibit the proliferation of PEDV CV777 and DY strain. The virus titer in chrysin and naringenin treatment groups at 24, 36, and 48 hpi were significantly lower than those of the virus control group (*p* < 0.01). And the inhibition effects were also observed at 60 h (*p* < 0.05). The inhibitory effects of ribavirin on PEDV at 36 h were significantly lower than that in the virus control (*p* < 0.05) (Figures 5A,B). In addition, chrysin and naringenin exhibited a significantly stronger inhibitory effect compared to ribavirin. The inhibitory effect of chrysin was the best, followed by naringenin and ribavirin.

### 3.5 Effect of chrysin and naringenin on prophylactic, viral entry and post-viral entry

**Figure 6:**
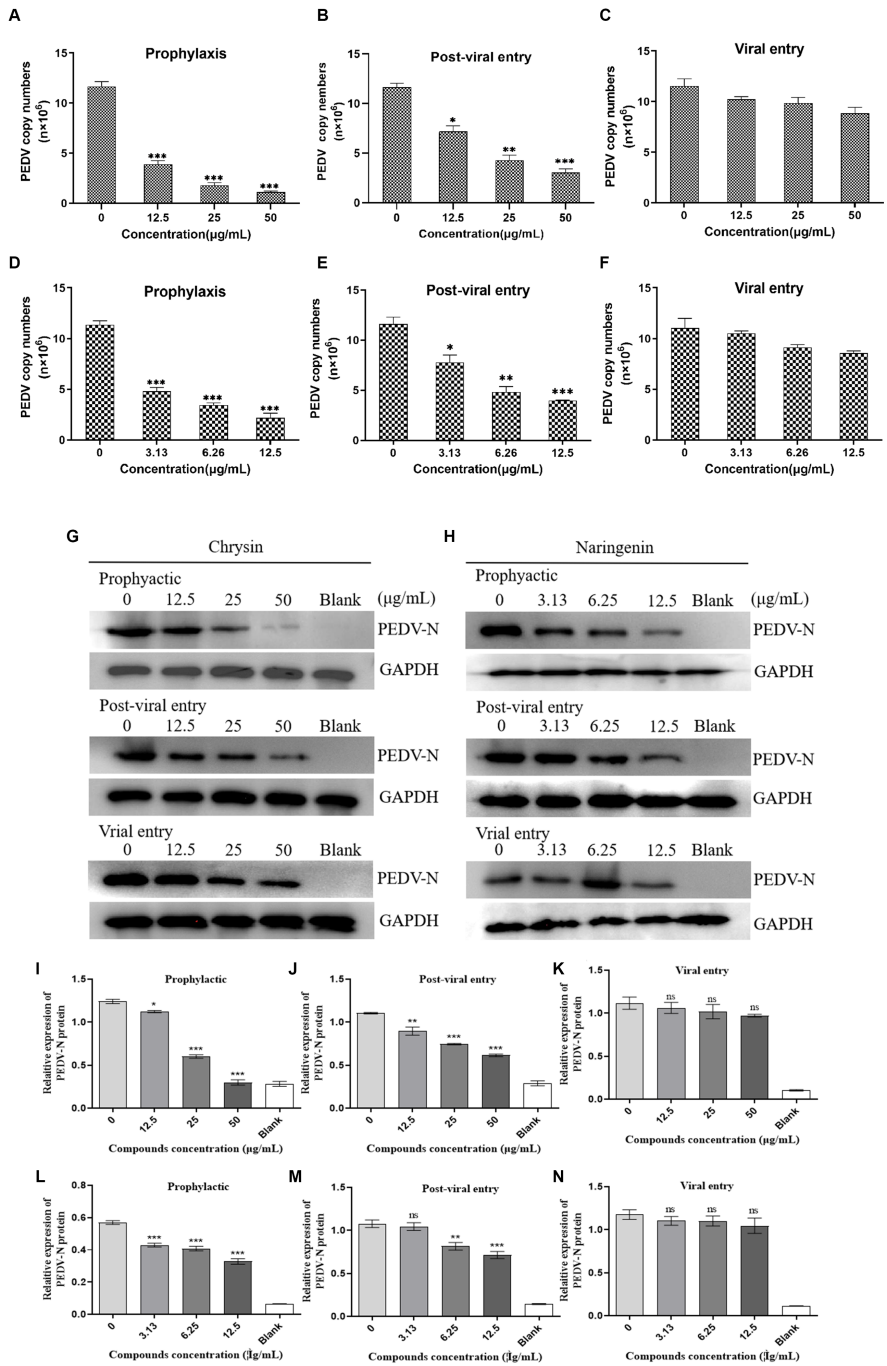
Effects of different action stages and concentrations of chrysin and naringenin on the virus. Dose-dependent inhibition of chrysin and naringenin on PEDV replication as examined by qPCR. **(A–C)** Chrysin. **(D–F)** Naringenin. The data from triplicate experiments are reported as means and SD of viral FIGURE 6 (Continued)copy numbers. **(D)** Western blot of N-protein in cells infected with PEDV and treated with chrysin and naringenin **(G)** Chrysin. **(H)** Naringenin. The internal reference gene GAPDH was used as an internal reference. Relative protein levels of PEDV-N in each group. Data are expressed as mean ± SD. **(I–K)** Chrysin. **(L–N)** Naringenin.

The qPCR detection results showed that chrysin and naringin had strong inhibitory activity in the prophylactic and post-viral entry stages. In prophylactic stage, the viral mRNA quantitation in the chrysin treatment groups with different concentrations were 3.87 × 10^6^ copies/μL (12.5 μg/mL), 1.78 × 10^6^ copies/μL (25 μg/mL), and 1.19 × 10^6^ copies/μL (50 μg/mL), all significantly lower than that of the virus control group (*p* < 0.001). The viral mRNA quantitation in the naringenin treatment groups were 4.79 × 10^6^ copies/μL (3.13 μg/mL), 3.21 × 10^6^ copies/μL (6.25 μg/mL), and 2.17 × 10^6^ copies/μL (12.5 μg/mL), all significantly lower than that of the virus control group (*p* < 0.001). In the post-viral entry stage, both compounds exhibited significant inhibitory effects on PEDV. The viral nucleic acid copy numbers in the drug treatment groups were significantly lower than those in the control group (*p* < 0.05). However, their inhibitory effect was slightly less pronounced compared to the prophylactic stage (Figures 6A,B,D,E). The immunoblotting results were basically consistent with the qPCR detection results. Chrysin and naringenin could significantly inhibit the expression level of PEDV N protein during the prophylactic and post-viral entry stage, and the inhibitory effect on the expression of PEDV N protein became more pronounced as the compound’s concentration increased. However, the expression level of the functional cellular structure protein GAPDH remained relatively stable (Figures 6G,H). The inhibitory effect was most pronounced when the concentration of chrysin was 50 μg/mL and naringenin was 12.5 μg/mL.

During the viral entry evaluation process, chrysin and naringenin extract did not show significant inhibitory effects, and their nucleic acid copy numbers were not significantly different from the control group (Figures 6C,F).

### 3.6 Molecular docking

**Figure 7:**
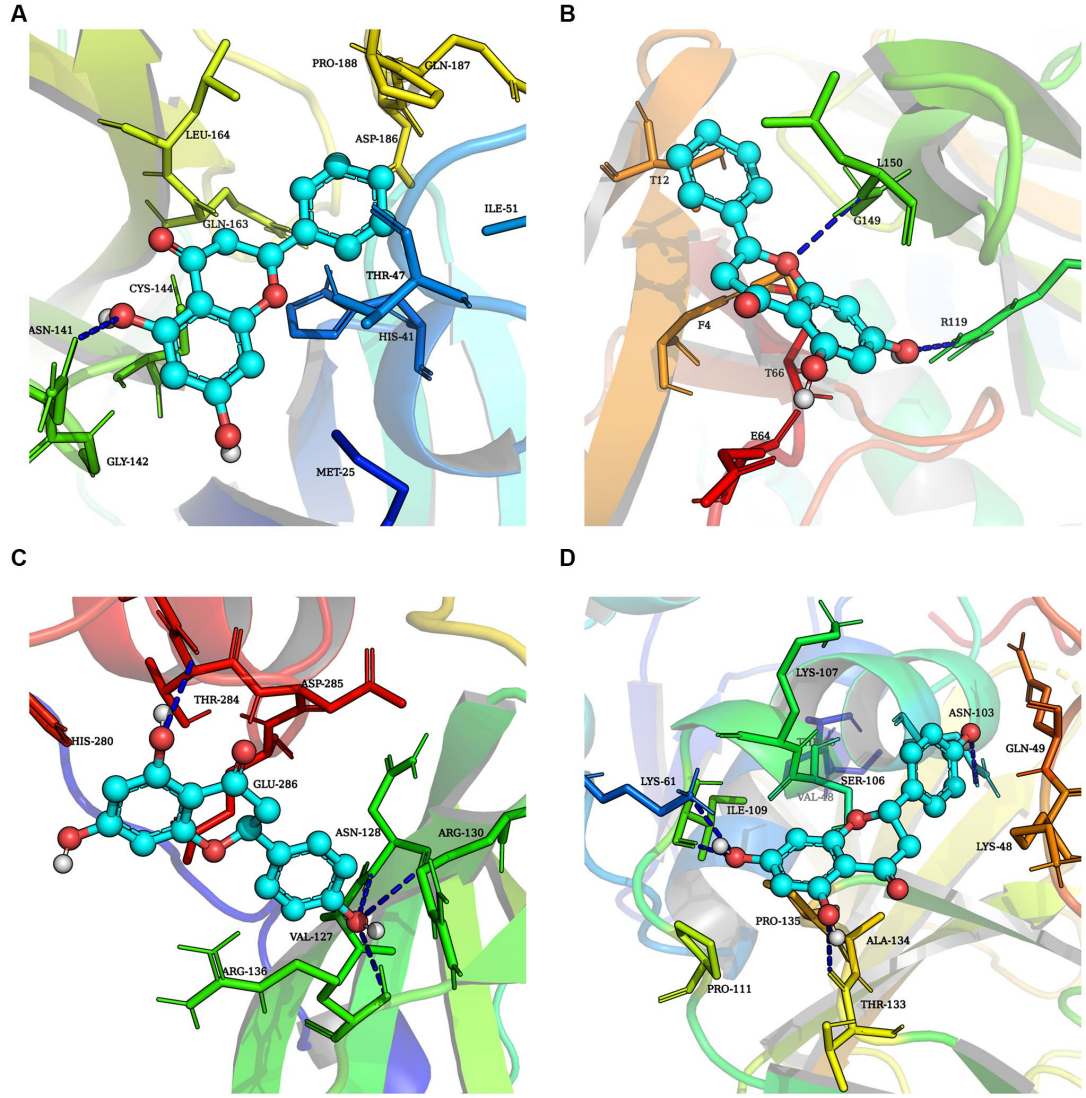
Molecular docking simulation of three natural compounds interacting with PEDV replication-related proteins. **(A)** Chrysin + 3CLpro. **(B)** Chrysin + PLP-2. **(C)** Naringenin + 3CLpro. **(D)** Naringenin + PLP-2.

This study performed molecular docking analysis between natural compound and replication-associated proteases (3CLpro and PLP-2 proteins) of PEDV (Figures 7A–D). The binding energies of chrysin to 3CLpro and PLP-2 proteins of PEDV are shown in Tables 2, 3. The results showed that the docking binding energies of chrysin and naringenin to the 3CLpro were −7.2 Kcal/mol and −7.4 kcal/mol, respectively, lower than the binding energies of 3CLpro original ligands. The binding energies of chrysin and naringenin with PLP-2 were −7.1 kcal/mol and −7.4 kcal/mol, respectively.

**Table 2:** Binding interactions of chrysin with the binding site and binding energy of protease and proteins of PEDV.

Protein/protease	PDB ID	Hydrogen bond interactions	Hydrophobic interaction	Binding energy (kcal/mol)	Binding energy of the original ligand (kcal/mol)
3CLpro	6L70	Arg103, Asn150, Arg294	Gly108, Glu109, Ser110, Glu201, Asn202, Thr288, Gly290	−7.2	−6.8
PLP-2	7F0U	Arg119, Leu150	Phe4, Thr12, Glu64, Thr66, Gly149	−7.1	/

**Table 3:** Binding interactions of naringenin with the binding site and binding energy of protease and proteins of PEDV.

Protein/protease	PDB ID	Hydrogen bond interactions	Hydrophobic interaction	Binding energy (kcal/mol)	Binding energy of the original ligand (kcal/mol)
3CLpro	6L70	Arg103, Glu201, Arg294	Gly108, Glu109, Ser110, Asn150, Thr288	−7.4	−6.8
PLP-2	7F0U	Lys61, Asn103, Ser106, Ile109, Thr133	Lys48, Gln49, Lys107, Pro111, Ala134, Pro135	−7.3	/

Hydrophobic and hydrogen bonds were the main forces between the two natural compounds and the viral proteins. Chrysin formed three hydrogen bonds, seven hydrophobic bonds with 3CLpro, two hydrogen bonds, and five hydrophobic bonds with PLP-2 (Tables 2, 3). The carbonyl oxygen atom on the pyran ring of chrysin and the phenol hydroxyl oxygen atom in the benzene ring are hydrogens bonded to the nitrogen atom on 3CLpro Arg103, and the benzene ring another phenolic hydroxyl oxygen atom was hydrogen bonded to the nitrogen atoms on Asn150 and Arg294 with bond lengths of 2.81 Å and 2.94 Å, respectively; the oxygen atom of the pyran ring was hydrogen bonded to the nitrogen atom of Leu150 of PLP-2 protein, and the phenolic hydroxyl group in the benzene ring was hydrogen bonded to the nitrogen atom of Arg119 with bond lengths of 3.24 Å and 3.01 Å, respectively.

**Figure 8:**
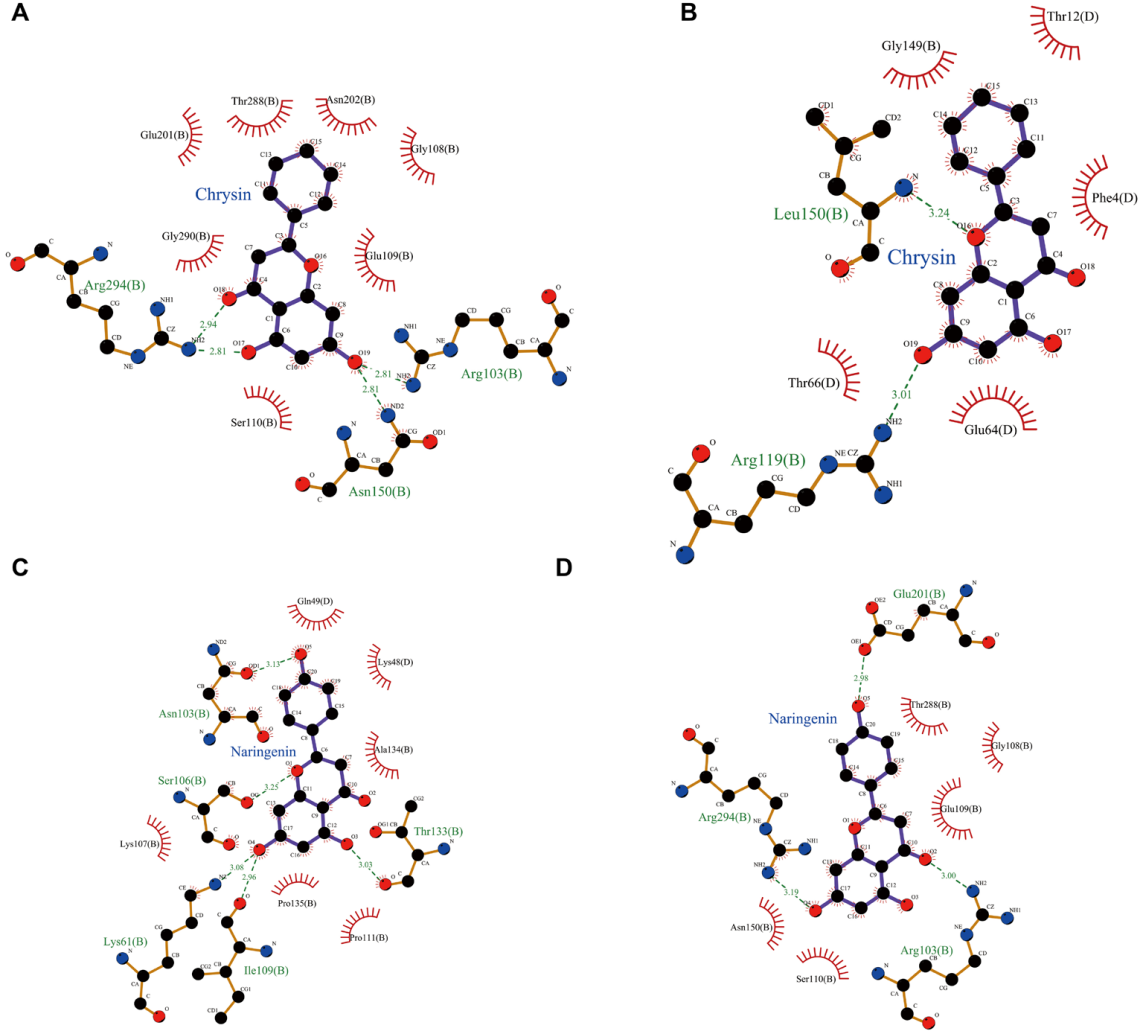
Schematic representation of hydrogen bond formation of two natural compounds interacting with PEDV-related proteins. **(A)** Chrysin + 3CLpro. **(B)** Chrysin + PLP-2. **(C)** Naringenin + 3CLpro. **(D)** Naringenin + PLP-2.

Naringin formed 3 hydrogen bonds and 5 hydrophobic bonds with 3CLpro; and 5 hydrogen bonds and 6 hydrophobic bonds with PLP-2. The carbonyl oxygen atom of the pyran ring, the phenol hydroxyl oxygen atom of the benzene ring attached to the pyran ring, and the phenolic hydroxyl oxygen atom on the other benzene ring were hydrogen bonded to Arg103, Glu201, and Arg294 of the 3CLpro protein with bond lengths of 3.00, 2.98, and 3.19 Å, respectively; the oxygen atom on the phenolic hydroxyl of the benzene ring was hydrogen bonded to Asn103 of the PLP-2 protein, the oxygen atom on the pyran ring was bonded to the Ser106 oxygen atom, and the oxygen atom on the phenolic hydroxyl of the phenyl ring attached to the pyran ring was hydrogen bonded to the nitrogen atom of Lys61, Ile109, and Thr133 oxygen atoms with bond lengths of 3.13, 3.08, 2.96, and 3.03 Å, respectively (Figures 8A–D).

The above results showed that the binding energy of chrysin and naringin to 3CLpro and PLP-2 proteins was higher. Meanwhile, the small molecules had high hydrogen bonding ability and hydrophobic interaction force with each protein, indicating that chrysin and naringenin had a good binding affinity with 3CLpro and PLP-2 and better binding activity with the target proteins.

### 3.7 Molecular dynamics studies

**Figure 9:**
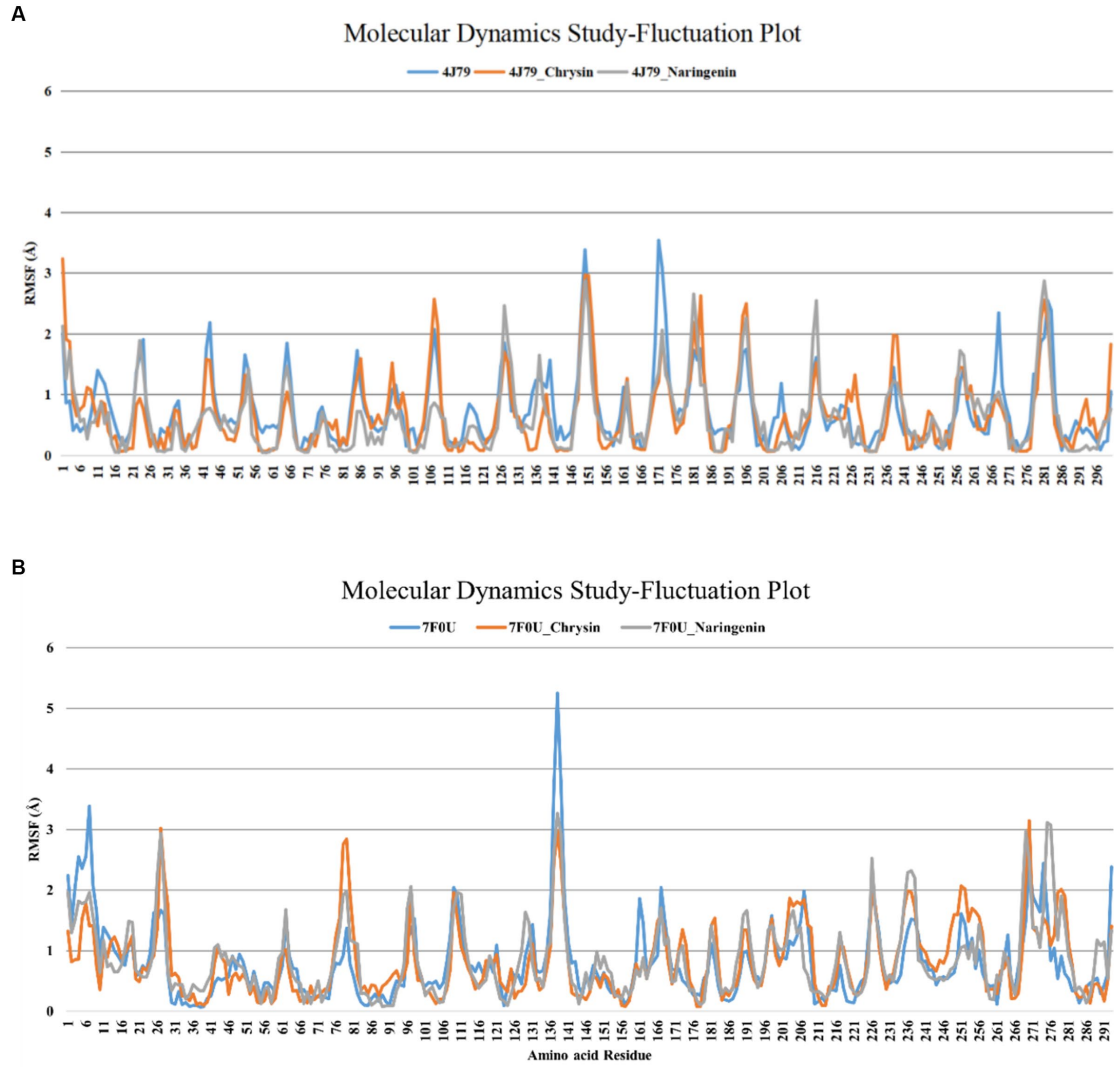
RMSF analysis of each protein complex of chrysin, naringenin, and PEDV. **(A)** 3CLpro; **(B)** PLP-2.

According to the analysis of RMSF, naringin had different degrees of binding ability with 3CLpro, and PLP-2 proteins. The mean RMSF values of chrysin with 3CLpro and PLP-2 protein were 0.89 Å, 0.83 Å, and 0.94 Å, respectively; the mean RMSF values of naringenin with 3CLpro and PLP-2 protein were 0.93 Å, 0.85 Å, and 0.86 Å, respectively. The results showed that the maximum RMSF values of complexes with replication-associated proteases was less than 3.5. Further analysis showed that the complexes of chrysin with 3CL protein showed peak fluctuations in 136–141 aa, 191–196 aa, 216–221 aa, 271–276 aa, etc.; the complexes with PLP-2 protein showed peak fluctuations in the regions of 25–31 aa, 76–81 aa, and 276–281 aa. The complexes with 3CL protein showed peak fluctuations in the regions of 136–145 aa, 238–242 aa, 216–221 aa, and 278–282 aa. The complex with PLP-2 protein showed peak fluctuations in the regions of 26–29 aa, 234–248 aa, and 272–276 aa (Figures 9A,B). Besides, no significant confirmation fluctuations were observed for other active sites. The above results indicated that the complexes formed by chrysin and naringenin with the two PEDV replication proteases had high stability. The RMSF waveform curves displayed a good fit with the original proteins. This further validates that Autodock Vina docking results are not obtained by chance.

## 4 Discussion

Chrysin and naringenin are two natural flavonoid compounds. Chrysin is the main active ingredient extracted from the purple wisteria plant and the Chinese red pine, both belonging to the family of *Fabaceae*, while naringenin is a secondary metabolite of citrus plants of *Rutaceae* (37). Both compounds have been confirmed to possess various biological activities, such as antioxidant (38), anti-inflammatory (39), anti-cancer (40), anti-asthmatic, anti-bacterial (41), and anti-arthritis effects (42), and they can enhance the host innate immune response (43). In recent years, domestic and foreign studies have found that chrysin and naringenin have good inhibitory effects on DNA and RNA viruses, including enterovirus 71, hepatitis virus, influenza virus, and dengue virus, providing theoretical references for the development of new antiviral drugs. For example, Wang et al. (44) found that chrysin and its 7-diisopropyl analogs could effectively inhibit the replication of enterovirus 71 by inhibiting 3CLpro protein activity, with an IC50 of 24.12 μM. It was also found that chrysin could inhibit HIV-1 gene expression at the transcriptional level by inhibiting casein kinase II. Moreover, chrysin exhibited good safety in animal tests, with no toxic effects on mice (45). Naringenin inhibited HBx-induced hepatic steatosis by reducing the transcriptional activity of SREBP1c, LXRα, and PPARγ in HBx-transgenic mice and HepG2 cells, indicating that naringenin may be a potential therapeutic drug for inhibiting virus-induced metabolic disorders (46). The above research indicates that chrysin and naringenin are two highly promising natural compounds with antiviral activity, which could provide a new direction for the development of novel antiviral drugs. However, there has been no report on the inhibitory activity and related mechanisms of these two compounds against coronavirus, which requires further exploration.

In our study, the anti-coronavirus activities of two natural flavonoid compounds were evaluated. The results showed that the SI index of chrysin and naringenin is significantly higher than the broad-spectrum antiviral drug ribavirin. In immunofluorescence assay (IFA) assay, the PEDV-specific green fluorescence was significantly reduced and weakened in both compound treatment groups. The qPCR and TCID_50_ assays also confirmed this conclusion, demonstrating that chrysin and naringenin had stronger inhibitory effects on PEDV compared to ribavirin.

In addition, natural compounds can act as antivirals at various stages of the virus life cycle, including attachment, replication, assembly, and release (47). Examining the antiviral effects of drugs at different stages can enhance our understanding of their mechanisms and serve as a reference for future clinical drug use. Current research has found that chrysin inhibits the early infection and replication of influenza virus A/PR/8/34 strain by activating the mammalian target of rapamycin (mTOR) to inhibit virus-induced autophagy and reduce viral titers in infected cells. As discussed by Zandi et al. (48) that different flavonoid compounds have different inhibitory effects on different proliferation stages of dengue virus (DENV-2). For example, naringenin exhibits direct virucidal activity against DENV-2, and treatment with 50 μg/mL naringenin after virus incubation can reduce DENV-2 RNA levels by 50%, but it does not affect the replication stage of DENV-2. Although chrysin and naringenin also have varying inhibitory effects on the hepatitis B virus (21), hepatitis C virus (49), and other viruses, but the stages of the virus life cycle targeted by these compounds are not yet clear. In this study, chrysin and naringenin showed strong inhibitory activity in the prophylactic and post-viral entry stages, with inhibitory effects on both the PEDV DY strain (field isolate) and the vaccine strain CV777. In prophylactic pattern, compounds are first incubated with cells, and then the cells are infected with virus. The results showed that the viral mRNA level and protein expression in drug treatment groups are all significantly lower than that of the virus control group (*p* < 0.001). This suggested that, on one hand, two natural flavonoids can penetrate the cell membrane and is still active intracellularly; On the other hand, it also implied that compounds may also affect the virus by binding to cell surface receptors to interfere virus attachment or entry. Similarly, both compounds also exhibited significant inhibitory effects in post-viral entry pattern. The significant role of this stage showed that drugs play a crucial part in inhibiting the life cycle activities of viruses after they invade cells. For example, drugs might affect viral genome replication, protein synthesis, assembly and release (21). Interestingly, the analysis of molecular docking results provides strong support for our hypothesis. In this study, the type and stability of the complexes formed by chrysin and naringenin with PEDV 3CLpro and PLP-2 protein were evaluated by using molecular docking and molecular dynamics simulation. The results showed that two compounds mainly bind to PEDV 3CLpro and PLP-2 protein through hydrogen bonding and hydrophobic interactions, and their binding energies were lower than those of the original ligands and PEDV-related proteases, indicating that chrysin and naringenin have a good binding affinity with the two proteins. And the complexes formed by the two natural compounds with PEDV proteases were highly stable, as indicated by the RMSF waveform curves fitting well with the original proteins and showing stable fluctuation trends. Current research confirmed that PEDV 3CLpro protein plays a crucial role in the replication of the virus by catalyzing the dimerization of the polypeptides pp1a (nsp4-nsp11) and pp1ab (nsp12-nsp16) to produce 13 non-structural proteins (50). The 3CLpro protein of various coronaviruses can inhibit the activation of the interferon signaling pathway by cleaving the important adapter molecule NF-κB in host cells (51). PEDV PLP2 has deubiquitination and deglycosylation activities, which can weaken the host innate immune response by antagonizing the expression of type I interferon, help the virus escape host immunity, and promote its replication and expression (52). Based on this, we believe that chrysin and naringenin may exert antiviral effects by interacting with virus 3CLpro protein, thereby affecting its role in the formation of PEDV non-structural proteins or alleviating its inhibitory effect on the expression of interferon in host cells. These two flavonoid compounds also might interfere with virus replication by inhibiting the activity of PLP2 protein, ultimately achieving an inhibitory effect on virus replication. Similar mechanism have also been confirmed in studies of other flavonoids’ effects on SARS-CoV and MERS-CoV (53).

However, research on the effects of chrysin and naringenin on PEDV is still very limited, and there are few reported natural compounds with anti-PEDV activity. Many potentially valuable compounds need further screening and research. Most studies on natural anti-PEDV compounds are still at the cellular level, and it is not yet clear whether they can achieve ideal effects in animals. The mechanism of action of existing natural compounds on PEDV is not fully understood. It is still unknown whether these natural compounds directly interact with the viral replication enzyme protein or other important structural proteins, blocking viral nucleic acid replication or adsorption to host cells. Further exploration is needed. In-depth studies on natural compounds’ anti-PEDV properties provide reliable technical means for preventing and treating porcine diarrhea and lay the foundation for the subsequent research on the mechanism of action of flavonoid compounds.

## 5 Conclusion

Both chrysin and naringenin displayed the most significant anti-PEDV activity by increasing the cell viability and decreasing the virus copy number. Both natural compounds could inhibit viral titer, mRNA and protein levels in prophylactic and post-viral entry stages of PEDV infection. Furthermore, chrysin and naringenin mainly interacted with viral replicase proteins such as 3CLpro and PLP-2 through hydrogen bonds and hydrophobic forces. The complexes formed by chrysin and naringenin with the two PEDV replication proteases had high stability.

This study lays the foundation for developing chrysin and naringenin as novel anti-PEDV therapeutic drugs.

## Data availability statement

The raw data supporting the conclusions of this article will be made available by the authors, without undue reservation.

## Ethics statement

Ethical approval was not required for the studies on animals in accordance with the local legislation and institutional requirements because only commercially available established cell lines were used.

## Author contributions

MG: Conceptualization, Data curation, Methodology, Writing – original draft. XX: Methodology, Writing – original draft. DC: Funding acquisition, Resources, Writing – review & editing. YR: Funding acquisition, Resources, Supervision, Writing – review & editing. YL: Methodology, Writing – review & editing. HX: Resources, Writing – review & editing. XL: Resources, Writing – review & editing. YZ: Methodology, Writing – review & editing. YM: Methodology, Writing – review & editing.

## Footnotes

^1^ https://www.uniprot.org/

^2^ https://pubch.em.ncbi.nlm.nih.gov/

^3^ http://openbabel.org/wiki/Main_Page
